# Ferroelectricity from Spontaneous Symmetry Breaking in Amorphous BN-Based Thin Films Grown via Atomic Layer Annealing

**DOI:** 10.1021/acs.cgd.6c00506

**Published:** 2026-06-10

**Authors:** Bipin Bhattarai, Dominic A. Dalba, Somayeh Saadat Niavol, Dilan M. Gamachchi, Indeewari M. Karunarathne, Xiaoman Zhang, Wangwang Xu, Dongmei Cao, Wen Jin Meng, Andrew C. Meng

**Affiliations:** † Department of Physics and Astronomy, 14716 University of Missouri, Columbia, Missouri 65211, United States; ‡ Department of Engineering and Industrial Professions, 5773 University of North Alabama, Florence, Alabama 35632, United States; § Department of Mechanical and Industrial Engineering, 5779 Louisiana State University, Baton Rouge, Louisiana 70803, United States

## Abstract

Boron nitride (BN) thin film growth typically requires high temperatures (>600 °C), which limits integration with patterned Si-based electronic devices. In this work, we demonstrate that atomic layer annealing (ALA) enables the growth of amorphous BN thin films at a relatively low temperature of 350 °C. Despite BN being centrosymmetric in its most stable hexagonal phase (h-BN), spontaneous symmetry breaking in the ALA-grown films results in a clear, switchable ferroelectric polarization, as confirmed by Positive-Up Negative-Down (PUND) measurements and a piezoresponse force microscopy (PFM) DC poling response consistent with ferroelectric behavior. X-ray photoelectron spectroscopy (XPS) and electron energy loss spectroscopy (EELS) show that, while the films are predominantly composed of boron and nitrogen, minor incorporation of carbon and oxygen potentially contributes to local symmetry breaking in the BN structure. Density functional theory (DFT) calculations show that external electric fields induce atomic displacement in h-BN, breaking centro-symmetry and leading to nonzero spontaneous polarization. These results highlight ALA as a viable low-temperature route for realizing functional BN thin films exhibiting emergent ferroelectricity for electronic device applications.

## Graphical abstract

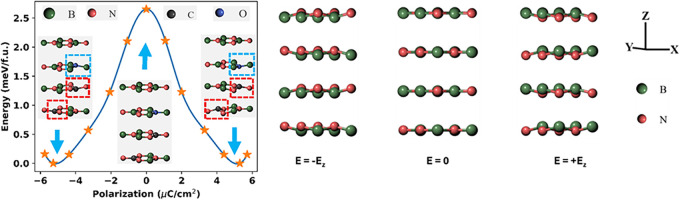

## Introduction

Boron nitride (BN) has garnered significant attention in recent years for advanced electronic devices. Its applications range widely, including low-k dielectrics with high breakdown fields, 2D electronic devices, capping layers, quantum materials, and more.^1–8^ Despite this, the vast majority of techniques used to grow BN involve high-temperature processing, often with temperatures in excess of 600 °C.^9,10^ To date, various deposition strategies have been developed for BN thin films targeting a range of applications, from thermal management and oxidation-resistant coatings to dielectrics in 2D electronics and quantum photonics.^11–14^ Most of these methods operate at elevated temperatures that are incompatible with complementary metal-oxide-silicon (CMOS) processes. For example, chemical vapor deposition (CVD), one of the most common approaches for large-area h-BN synthesis, generally requires growth temperatures between 750–1100 °C.^15,16^ Molecular beam epitaxy (MBE)^17,18^ and pulsed laser deposition (PLD)^19,20^ typically operate at >700 °C, while atomic layer deposition (ALD), though more thermally forgiving, still often exceeds 400 °C depending on the precursors used and growth procedure.^21,22^

A major difficulty arises due to the high decomposition temperatures of common boron precursors such as triethylborane or trimethylborane.^23^ While boron precursors with lower decomposition temperature exist, e.g., trimethoxyborate (TMOB),^24^ they can lead to defects and substantial carbon and oxygen incorporation. On the other hand, process temperatures above 400 °C are incompatible with back-end-of-line (BEOL) CMOS fabrication. While there is demand for the various functionalities afforded by BN in electronic devices, a low-temperature BEOL-compatible growth process opens the door to a realistic path toward integration of BN with advanced electronic devices.

In this study, we demonstrate the growth of amorphous BN films using a low-temperature (350 °C) atomic layer annealing (ALA) process.^25,26^ ALA is a variant of ALD that involves an additional plasma pulse in addition to the precursor pulses used to promote crystallization and growth. Our BN growth process uses triethylborane (TEB) and hydrazine (N_2_H_4_) precursors and a N_2_ inductively coupled plasma (ICP). ALA provides a potentially scalable CMOS-compatible process for BN integration in Si-based electronic devices. Elemental composition in the films measured using X-ray photoelectron spectroscopy (XPS) and electron energy loss spectroscopy (EELS) in the Scanning/Transmission Electron Microscope (S/TEM) showed minor incorporation of carbon and oxygen. Furthermore, high-resolution TEM (HRTEM) imaging showed the films to be amorphous.

While measuring the electrical properties of amorphous ALA BN films, we unexpectedly observed an emerging ferroelectric response. To our knowledge, this has not been previously reported. We note that ferroelectricity has been previously observed in BN-based thin films due to various types of symmetry breaking: engineered stacking sequences,^27–30^ extrinsic atom incorporation,^31,32^ and the wurtzite crystal structure.^33^ There is also both experimental precedent and a theoretical framework grounded in physics that explains ferroelectricity in amorphous materials through ordered clusters of permanent dipoles.^34,35^ We rationalize the observation of ferroelectricity in amorphous BN-based films, in several considerations: (1) the wurtzite structure polymorph of crystalline BN is both polar and noncentrosymmetric, with multiple examples of isostructural materials exhibiting ferroelectricity;^36–40^ (2) extrinsic alloying-induced asymmetry is known to induce ferroelectricity in BN; (3) ferroelectricity has been previously observed in amorphous nitride-based thin films;^41^ and (4) ferroelectric switching in wurtzite-based ferroelectric materials occurs through atomic displacements that move between wurtzite structures of opposite polarity through the nonpolar h-BN structure.^42^

In this study, we demonstrate the experimental observation of ferroelectricity in amorphous ALA BN-based thin films through positive-up-negative-down (PUND) electrical measurements and piezoresponse force microscopy (PFM) DC bias poling experiments. Ferroelectric behavior of BN-based films remains relatively underexplored but holds significant promise given the wide range of functionalities afforded by this class of materials. To provide a physics-based rationale of ferroelectricity in amorphous BN-based thin films, we perform DFT calculations showing that external electric fields result in the atomic displacement of h-BN from trigonal to tetrahedral coordination. We observe a nonzero spontaneous polarization in the resulting distorted wurtzite structure, consistent with ferroelectricity.

## Experimental Section

To explore low-temperature growth of BN thin films with potential BEOL compatibility, we designed an ALA process using TEB and N_2_H_4_ ^41,43^ as the boron and nitrogen precursors, respectively. N_2_H_4_ was chosen over more conventional nitrogen precursors such as ammonia due to its higher reactivity at low temperatures, which promotes the formation of nitrogen-rich films under reduced thermal budgets.^41^ ALA was performed in an Anric Technologies AT650P plasma ALD system equipped with a remote ICP. The deposition was performed at a substrate temperature of 350 °C. The ALA process consisted of supercycles of 3 pulses: (1) a 30 ms pulse of TEB (5 s prolonged exposure) followed by a 30 s nitrogen (N_2_) purge; (2) a 30 ms pulse of N_2_H_4_ followed by a 60 s N_2_ purge; (3) a 23 s N_2_ ICP plasma (100 W RF power) pulse followed by a 17 s N_2_ purge. This sequence resulted in a total cycle time of about 135 s. The details of the process are shown in Figure 1a. The TEB and N_2_H_4_ precursors were introduced by passive vapor diffusion from sealed source cylinders without the use of a carrier gas, relying solely on their respective vapor pressures. The purging gas (N_2_) flow rate was fixed at 5 sccm throughout the entire growth process. Although no oxygen-containing precursors were intentionally introduced, minor oxygen incorporation is expected due to the reactor’s base pressure (∼6 mTorr), which may allow trace amounts of oxygen or moisture into the process. Film thicknesses were measured using variable-angle spectroscopic ellipsometry (J.A. Woolam VB-400, 75 W light source, HS-190 monochromator), and the resulting growth rate was 1.03 Å/cycle, as depicted in Figure 1b. Notably, no film growth is observed when the deposition is carried out without the N_2_ plasma pulse, demonstrating that purely thermal ALD BN deposition is not achievable at the low temperature of 350 °C under the present conditions.

**Figure 1:**
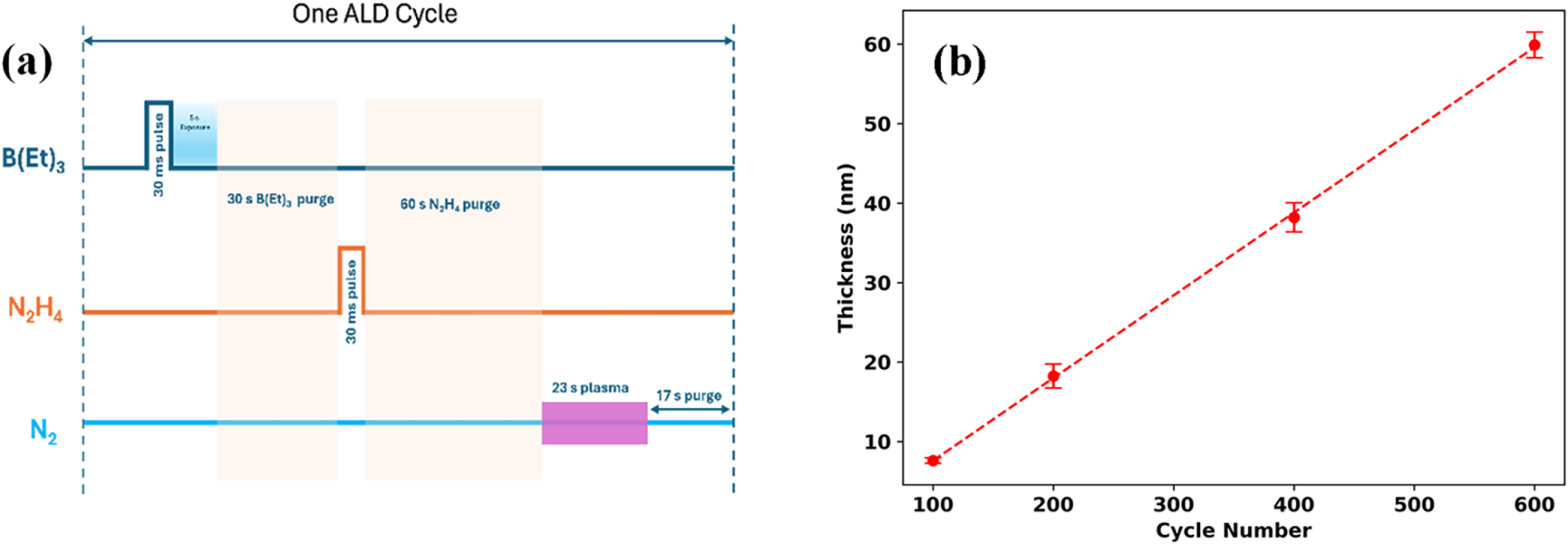
(a) Schematic of the ALD cycle used for BN thin film growth using triethylborane (B­(Et)_3_) and hydrazine (N_2_H_4_) as precursors, (b) film thickness of BN as a function of ALD cycle.

XPS depth profiles were measured using a Scienta Omicron ESCA 2SR X-ray Photoelectron Spectroscope with a monochromatic Al Kα excitation source. XPS spectra were acquired from BN films as-grown and after sputter etching with a 4.5 kV, 2 μA Ar^+^ ion beam for 0–120 min. Data processing was performed using the CasaXPS software. Cross-section TEM sample fabrication was performed using a ThermoFisher Helios Hydra plasma-focused ion beam/scanning electron microscope (PFIB/SEM). The sample was protected from the ion beam during the lift-out process through electron beam-induced deposition of W, followed by Xe^+^ ion beam-induced deposition of W. While the initial lift-out was performed using Xe^+^ ions, subsequent thinning at successively lower accelerating voltages was performed using Ar^+^ ions, with final thinning carried out at 2 kV. Structural characterization using S/TEM was performed using a probe aberration-corrected ThermoFisher Spectra 300 instrument. Imaging in both high-resolution TEM (HRTEM) and STEM imaging modes was performed at 300 kV accelerating voltage. Electron Energy Loss Spectroscopy (EELS) spectra were collected using a Gatan EF-CCD camera at a 37 mm camera length using a 5 mm Gatan imaging filter (GIF) aperture to yield a collection angle of 100 mrad.

Pt top electrodes (120 nm thick, 250 μm radius) were sputtered through a shadow mask (Stencils Unlimited) to fabricate capacitor devices; the degenerately doped Si wafer was used as the bottom electrode. Positive-Up Negative-Down (PUND) measurements on capacitor devices using a Keithley 4200A-SCS Parameter Analyzer were performed to evaluate the polarization properties of BN thin films.^41,44^ The input voltage waveform consisted of trapezoidal pulses with 10 μs rise, hold, and fall times (vide infra Figure 4a). An initial reset pulse of negative polarity was applied to align randomly oriented dipoles. Two subsequent pulses (P, U) of positive polarity are then applied to probe the forward total (polarization switching and leakage) and leakage currents, respectively. The second pulse induces only leakage current because the polarization has already been switched by the first pulse. Two more pulses (N, D) of negative polarity are then applied to probe the reverse total (polarization switching and leakage) and leakage currents, respectively. The forward and reverse polarization currents are calculated as (P–U) and (N–D), integrated over the pulse duration to obtain charge, and divided by the top electrode area to obtain polarization.

A Bruker Dimension Icon atomic force microscope (AFM) equipped with the PFM module was used to perform PFM DC poling experiments. Piezoresponse amplitude and phase were measured using a 10 V drive amplitude with a 7 kHz ac bias wave. Poling was performed by applying a DC bias of +25 V and −25 V in a square-in-square geometry to the AFM tip with respect to the grounded sample chuck during a PFM scan with a 100 mV ac bias. Residual changes to piezoresponse were measured by performing PFM measurements of a larger area containing the DC poled regions.

## Results and Discussion

Figure 2a depicts the XPS depth profile of the film. While boron and nitrogen are the major constituents of the film, with a B:N ratio of approximately 2:1, minor incorporation of carbon and oxygen is observed. We hypothesize that the presence of carbon is due to incomplete decomposition of TEB at 350 °C. Here, we observe that the C composition slightly increases as the growth proceeds to larger film thicknesses. This is consistent with decreasing C content with increasing plasma exposure. High-resolution XPS core-level spectra of the BN film, obtained after 20 min of Ar sputtering, are presented in Figure 2b–e. The key spectral features correspond to the 1s core levels of boron (B), nitrogen (N), carbon (C), and oxygen (O), appearing at binding energies of approximately 191, 398, 284, and 533 eV, respectively. The observed binding energies for B 1s and N 1s are in good agreement with the characteristic values for h-BN, typically reported as 190.6 and 398.2 eV, respectively.^45^

**Figure 2:**
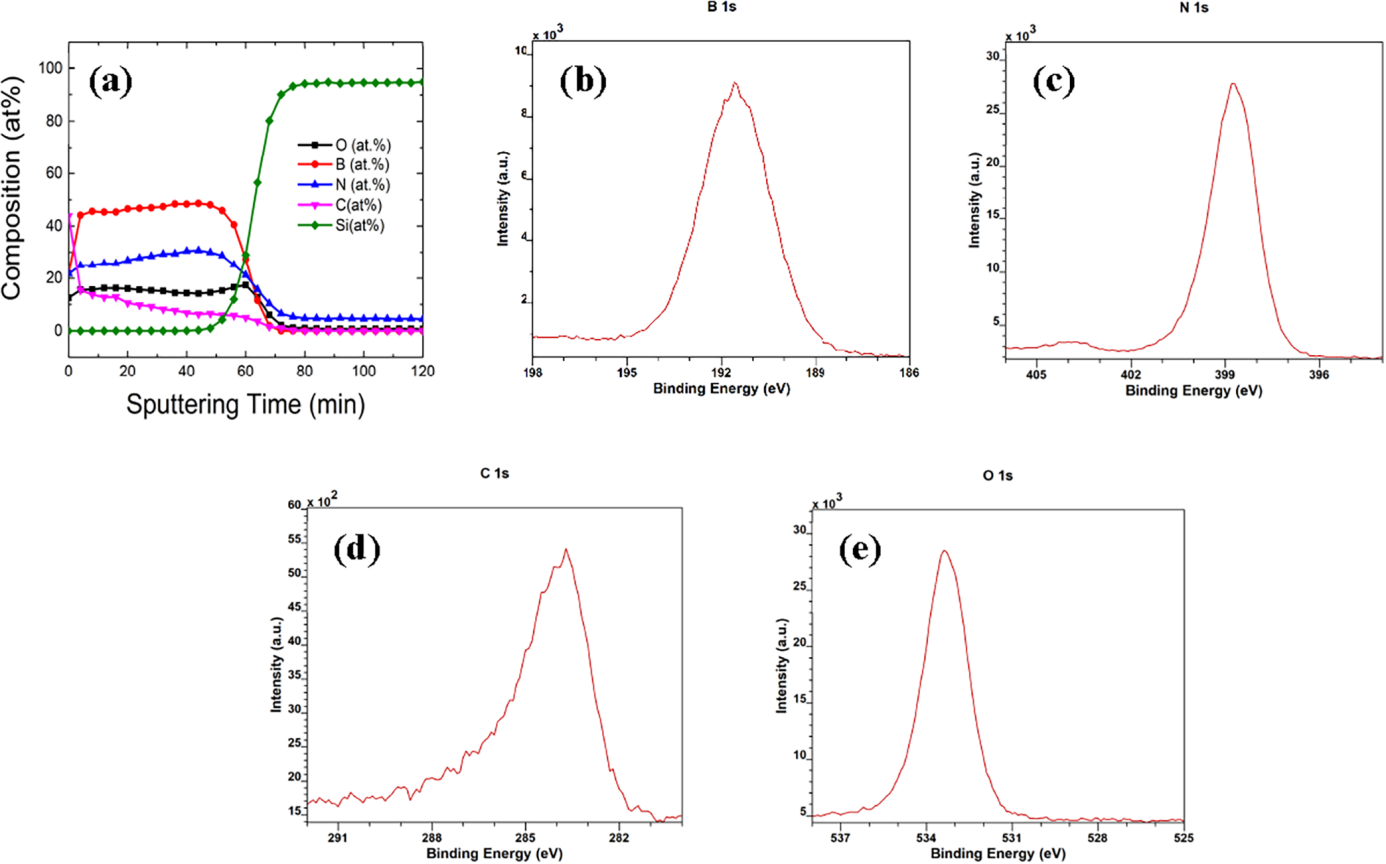
(a) XPS depth profile and detailed XPS core level scans of: (b) B 1s, (c) N 1s, (d) C 1s, and (e) O 1s signals.

Structural characterization using S/TEM is shown in Figure 3. Cross-section HRTEM imaging (Figure 3a) in the (110) zone axis of the Si (001) substrate shows phase contrast consistent with the ALA BN film being amorphous. The fast Fourier transform (FFT) (Figure 3a, inset) shows diffuse ring features consistent with an amorphous material. EELS mapping (Figure 3c–f) was performed in the red rectangular region of interest in the high-angle annular dark field (HAADF) STEM image (Figure 3b). The EELS spectrum of the sample (Figure 3g) exhibits several distinct features over a wide energy range. The B K-edge displays two prominent loss features at 192 and 199 eV, arising from transitions of B 1s electrons into unoccupied π* and σ* states, respectively.^46^ The C K-edge shows features at approximately 286 and 294 eV, where the peak near 286 eV corresponds to the C 1s → π* transition of sp^2^-bonded carbon, and the higher-energy feature at 294 eV is attributed to the C 1s → σ* transition, indicating a modest contribution from sp^3^-bonded carbon environments.^47^ Similarly, the N K-edge exhibits two distinct features at approximately 401 and 409 eV, corresponding to N 1s → π* and σ* antibonding transitions, respectively.^48^

**Figure 3:**
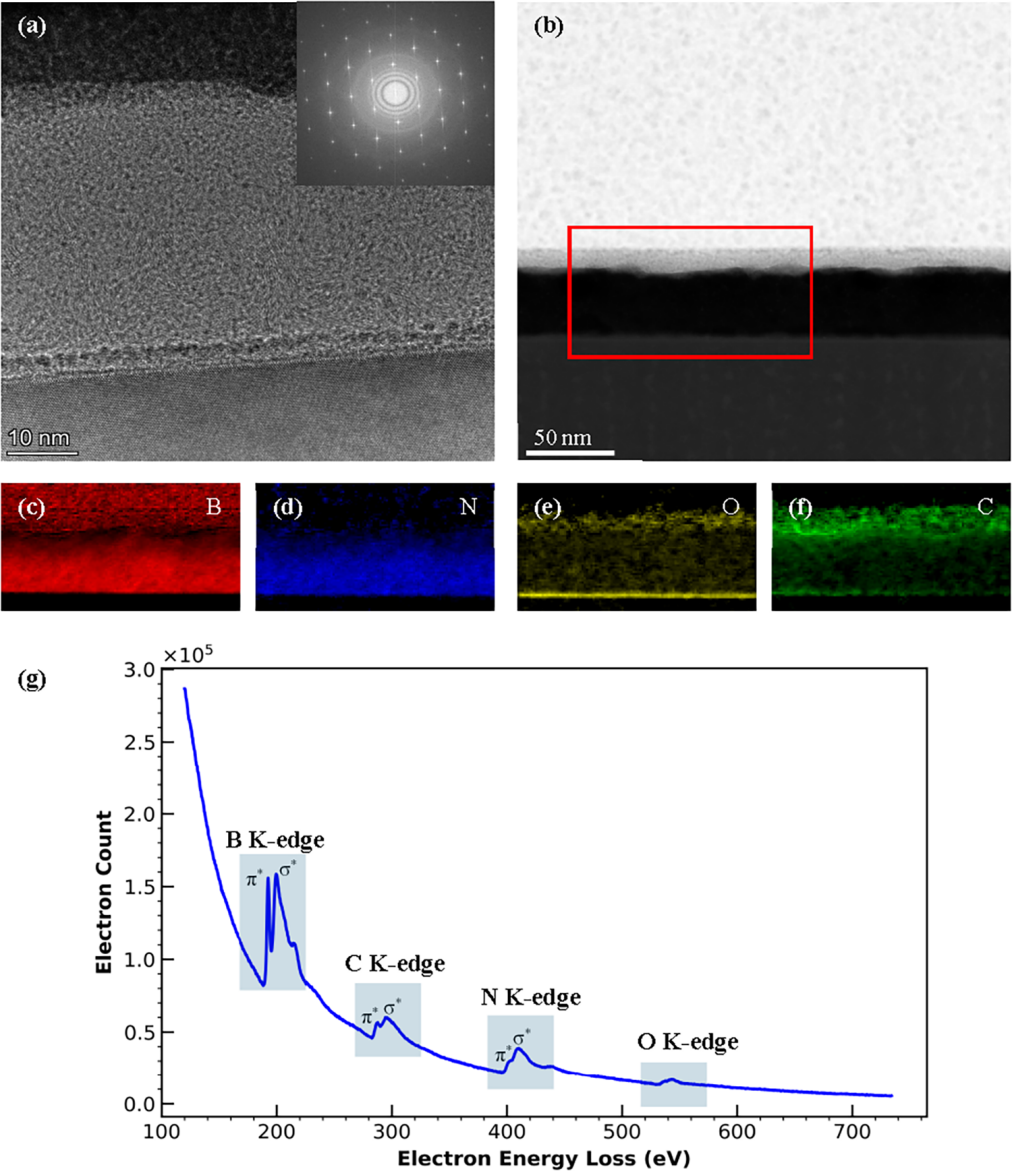
(a) Cross-sectional HRTEM image of the BN film on Si (001) along the Si ⟨110⟩ zone axis; inset: FFT displaying diffuse rings characteristic of amorphous material. (b) HAADF-STEM image of the same region; the red box marks the area used for EELS analysis. (c–f) EELS elemental maps from the selected region showing the spatial distribution of the film constituents. (g) Representative EELS spectrum acquired from the BN layer.

Figure 4a shows a typical PUND measurement obtained by poling the device at voltages ranging from 14 to 20 V, corresponding to electric fields of 3.9–5.56 MV/cm. The solid lines show the applied voltage waveform, and the dotted lines represent the current response. We observe for each applied voltage pulse train that the magnitude of the current in the P and N pulses is greater than that in the U and D pulses, implying a nonzero switchable polarization in the film induced by the electric field. The forward and reverse polarizations are obtained from (P–U) and (N–D) (Figure 4b). A forward polarization current of ∼1.5 mA is obtained when the device is poled at 20 V, whereas the corresponding reverse polarization current is ∼1.0 mA. Similar asymmetry in the forward and reverse polarization currents is observed at different voltages. This effect is sometimes observed in wurtzite nitride-based ferroelectric materials but could also arise from our devices having different top (Pt) and bottom (degenerately doped Si) electrode materials. Leakage corrected polarization–electric field (*P*–*E*) hysteresis loops obtained from the PUND data at room temperature are presented in Figure 4c. This data only shows the switching contribution to the polarization current. Leakage corrected P-E data is obtained from the PUND measurement – integrating the P and N pulses gives the combined forward and reverse polarization and leakage currents, respectively; integrating the U and D pulses gives the forward and reverse leakage currents, respectively. Thus, the forward and reverse switching polarizations are obtained from the integrals of P–U and N–D divided by the device area. The electrical leakage characteristics of the devices were also investigated. As a representative example, the leakage behavior of the devices poled at 18 V (5 MV/cm) is shown in Figure 4d. The leakage current density shows an excellent fit to the Poole–Frenkel emission model (*R* ^2^ = 0.998), indicating that the dominant leakage conduction mechanism arises from charge-carrier hopping through defect states. Such behavior is commonly observed in nitride-based ferroelectric materials.^49^

**Figure 4:**
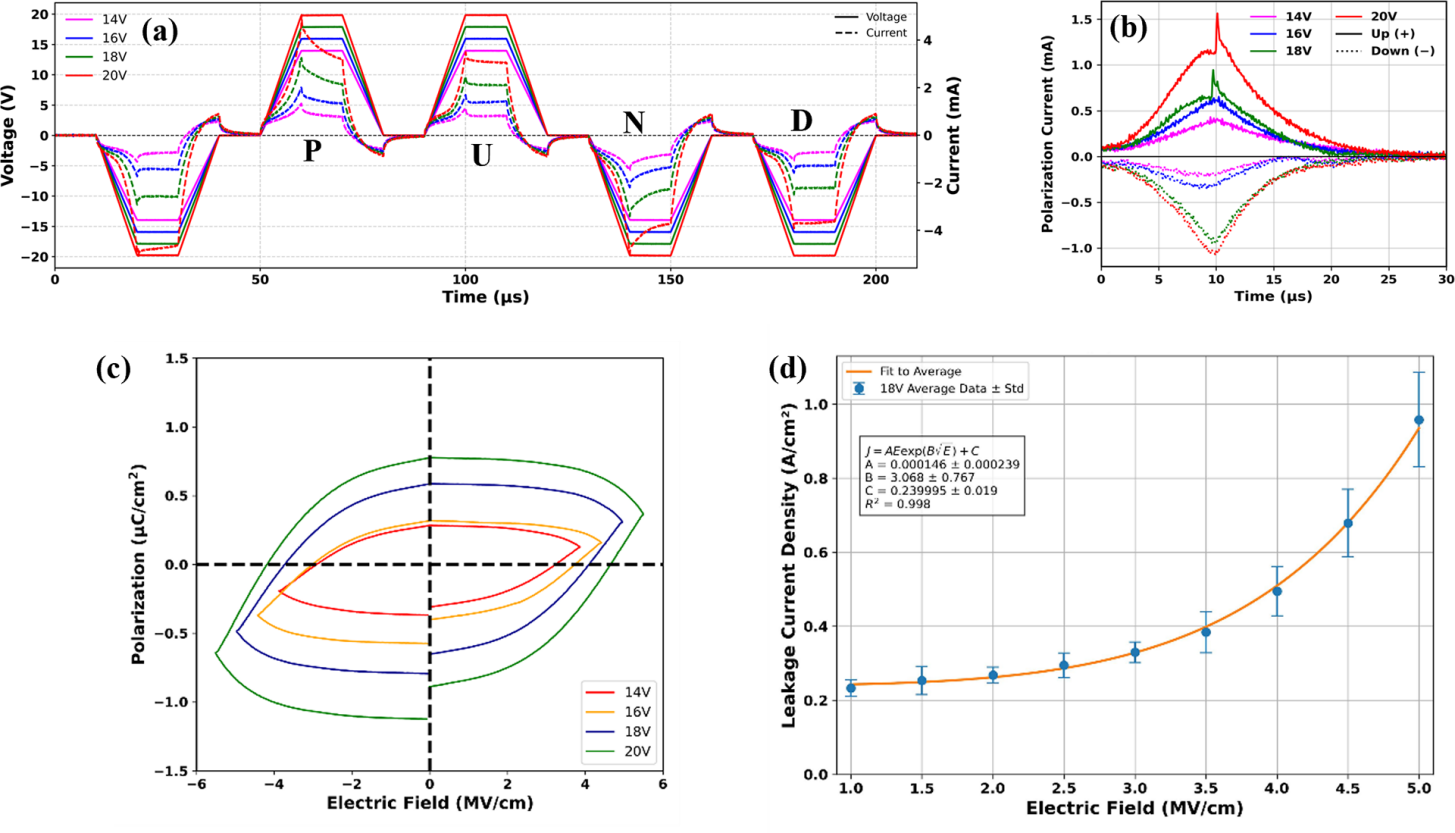
(a) PUND measurement of the film, (b) polarization current obtained from P–U (positive) and N–D (negative) pulse subtraction, (c) P-E hysteresis loops extracted from PUND measurement, and (d) fit of the leakage current density to Poole–Frenkel equation.

To understand a potential origin of the switchable polarization observed in the film, we performed DFT calculations (for details, see Supporting Information) of energy and atomic displacement in h-BN as a function of external electric field. This is done by performing a cell relaxation under the nonzero electric field. In a single layer of h-BN, coplanar B and N atoms are trigonally coordinated; however, the application of an out-of-plane (*c*-axis) electric field induces an out-of-plane (*c*-axis) displacement between B and N atoms that is correlated with the applied electric field strength. The resulting structure is a distorted wurtzite structure with tetrahedral coordination between B and N atoms. The symmetry-broken structure exhibits a nonzero polarization that we calculate using the Berry Phase method (Figure 6a). The resulting energy landscape for switching as a function of polarization as a reaction coordinate and crystal structures showing atomic displacements in the presence of an electric field applied along ±*z*-direction are shown in Figure 6a,b. As shown in Figure 6b, the external applied electric field along the *z*-direction breaks the centro-symmetry of the system, resulting in structures with nonzero switchable polarization.

We can rationalize this as a difference in the response of cation and anion cores under external electric fields. Furthermore, we calculate the spontaneous polarization of the distorted wurtzite structure of BN caused by electric field-induced symmetry breaking in h-BN using the Berry phase method^38^ at different values of the applied external electric field (Figure 5a). Compared to the experimentally observed remanent polarizations (Figure 5b), as measured by PUND, the DFT calculated spontaneous polarizations (Figure 5a) are slightly lower in magnitude: for example, at an applied electric field of 5 MV cm^–1^, the calculated polarization of h-BN is approximately 0.42 μC cm^–2^, whereas the experimentally measured polarization of the film reaches around 0.8 μC cm^–2^ (2*P*_r_ ≈ 1.6 μC cm^–2^). This suggests that other factors (e.g., extrinsic alloying with C and O) also contribute to symmetry-breaking-induced ferroelectricity in the amorphous ALA BN films. For example, highly electronegative O atoms can locally induce dipoles (see Figure 6).

**Figure 5:**
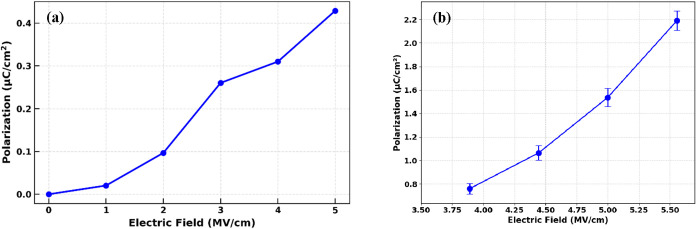
(a) DFT-based calculation of electric polarization in h-BN and (b) polarization (2*P*_r_) of the thin film obtained from PUND measurements.

**Figure 6:**
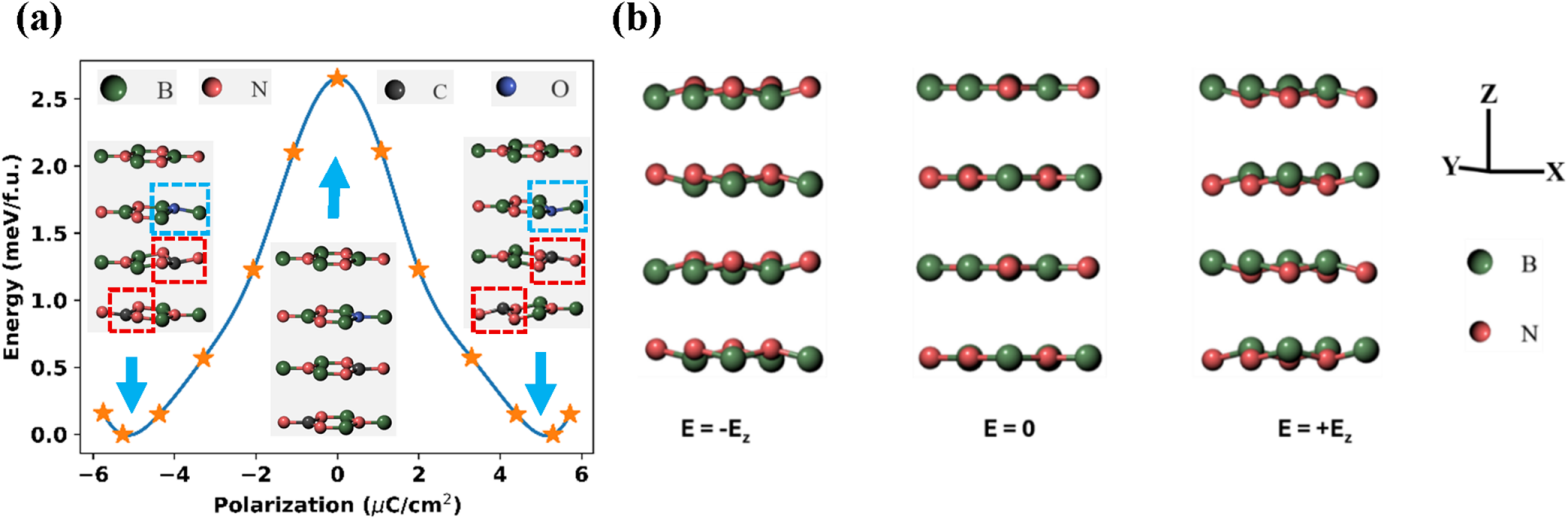
(a) Energy as a function of polarization in B­(OC)N with symmetry-broken h-BN structure; (b) atomic displacements of B and N atoms in the presence of an external electric field in out-of-plane (*z*) direction.

Ferroelectric behavior in the BN thin films was further examined using PFM DC poling experiments. Spatially localized DC bias poling was performed by applying +25 V (outside dotted square) and −25 V (inside dotted square) in a square-in-square geometry (Figure 7). Following poling, these regions show significant changes in surface height (Figure 7a,d), and observable differences in piezoresponse amplitude (Figure 7b,e) and faint differences in piezoresponse phase (Figure 7c,f). While piezoresponse can exist in materials that are piezoelectric but not ferroelectric, electric-field-induced strains and polarizations cannot be sustained in those materials in the absence of an applied external electric field. Thus, any residual change in height and piezoresponse amplitude/phase indicates an electric-field-induced change in strain and polarization that is sustained even when the electric field is removed, which is consistent with ferroelectricity.

**Figure 7:**
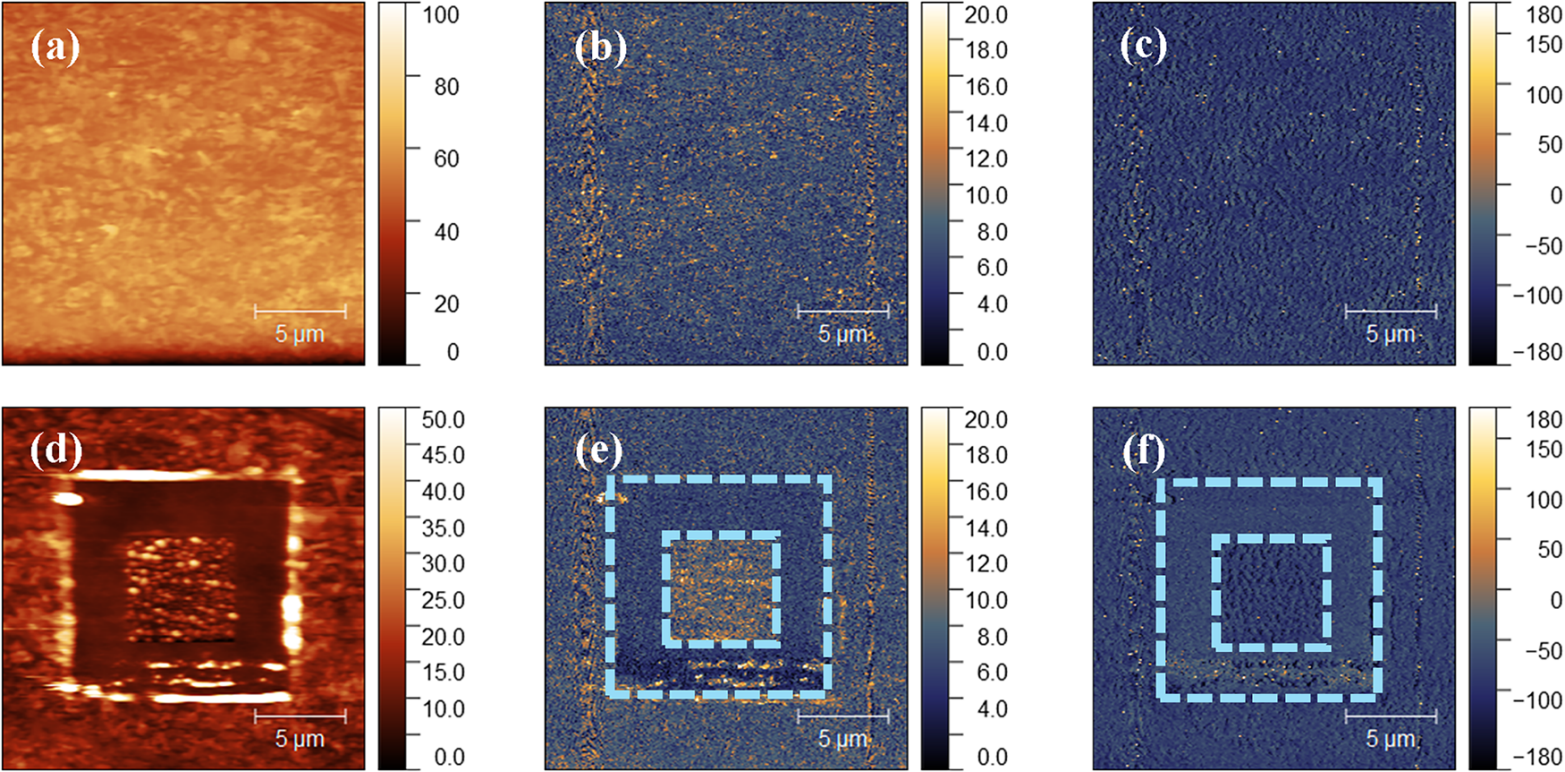
Piezoresponse force microscopy (PFM) analysis of the BN film before and after electrical poling. (a, d) Height maps, (b, e) amplitude maps, and (c, f) phase maps, captured before (top) and after (bottom) DC bias poling, respectively.

One unique advantage afforded by the ALA process is the capability of growing BN-based thin films using the commonly used TEB precursor at BEOL-compatible temperatures. We believe this point alone significantly improves the outlook for BN integration with advanced electronic devices. BN growth typically requires very high temperatures: many common boron precursors have high decomposition temperatures; BN is also very chemically inert, so there is a high energetic barrier to its formation.^9^ Our observation that no growth occurs in the absence of the plasma annealing step of the ALA process indicates that the activated species generated by the remote ICP is the key to low-temperature BN deposition. The unexpected observation of emergent ferroelectricity in amorphous ALA BN films is representative of the many opportunities to explore untapped emergent physics in BN-based materials.^1–8^

## Conclusions

In this study, we developed an ALA growth process for amorphous BN-based thin films at BEOL-compatible temperatures. This presents a significant opportunity for integration of BN-based thin films into advanced Si-based electronic devices at a critical juncture where novel materials and functionalities are in high demand. Compositional analysis based on XPS and EELS measurements indicates that the films are primarily composed of B and N, with minor incorporation of C and O. Spontaneous symmetry breaking induced by external electric fields and extrinsic alloying in these amorphous ALA BN-based thin films gives rise to a measurable ferroelectric response, as confirmed by PUND measurements and PFM DC bias poling. BN is already well-established as a wide-band gap, insulating material with wide-ranging functionality in transistor applications, particularly as a gate dielectric and interfacial layer.^50–52^ The emergence of polarization switching in BN-based films grown at low temperatures suggests that such structural modifications may extend the functional scope of BN beyond its conventional role, offering opportunities for added functionality within electronic device architectures while remaining compatible with CMOS thermal constraints.

## Supplementary Material



## Notes

Additional details for DFT simulations, including variable cell relaxation (vc-relax), relaxation (relax), self-consistent field (scf), and non-self-consistent field (nscf) calculations. (PDF)
