# Supplementary material for: Ferroelectricity from Spontaneous Symmetry Breaking in Amorphous BN-Based Thin Films Grown via Atomic Layer Annealing
Source: Cryst Growth Des. 2026 Jun 10;26(13):5157–65. doi: 10.1021/acs.cgd.6c00506 (PMC13329899; doi:10.1021/acs.cgd.6c00506)
Supplement: Supplementary file 1 [file cg6c00506_si_001.pdf]

Ferroelectricity from spontaneous symmetry breaking in amorphous BN-based thin films grown via atomic layer annealing

Bipin Bhattarai,[1] Dominic A. Dalba,[1] Somayeh Saadat Niavol,[1] Dilan M. Gamachchi,[1] Indeewari M. Karunarathne,[1] Xiaoman Zhang,[2] Wangwang Xu,[3] Dongmei Cao,[3] W.J. Meng,[3] Andrew C. Meng[1]

[1]*Department of Physics and Astronomy, University of Missouri, Columbia, MO 65211, USA. E-mail: acmeng@missouri.edu*

[2]*Department of Engineering and Industrial Professions, University of North Alabama, Florence, AL 35632, USA.*

[2]*Department of Mechanical and Industrial Engineering, Louisiana State University, Baton Rouge, LA 70803, USA*

DFT Details

All density functional theory (DFT) calculations were performed using the Quantum ESPRESSO package[1–3]. A 32-atom 2×2×2 sized supercell was used in the calculation. The calculations were carried out sequentially. First, the hexagonal boron nitride (h-BN) structure was optimized using a variable cell relaxation (vc-relax) calculation in the absence of an external electric field. Subsequently, a relaxation calculation (relax) was then performed in the presence of a uniform electric field applied along the *c*-axis (lelfield = .true.) to obtain the equilibrium atomic positions under a nonzero applied electric field. The c-axis polarization is obtained by performing a self-consistent field (scf) calculation to obtain the converged charge density followed by a non-self-consistent field (nscf) calculation for the polarization using the Berry phase approach[4,5] (lberry = .true.).

Projector augmented-wave (PAW) pseudopotentials were employed to describe the core-valence electron interactions. A plane-wave energy cutoff of 80 Ry was used, with the total energy convergence threshold set to 10^{-7}Ry. Brillouin-zone integrations were performed using an 12×12×8 Monkhorst-Pack *k*-point mesh. The exchange–correlation effects were treated within the generalized gradient approximation (GGA) using the Perdew-Burke-Ernzerhof (PBE) functional [6]. All relaxed structures satisfied a Hellmann-Feynman force criterion of less than 0.5 meV/Å

To demonstrate bi-stable polarization states, we considered a 32-atom 2×2×2 h-BN supercell containing O and C impurity atoms, chosen to mimic the experimental compositions. We then

calculate energy as a function of polarization as a reaction coordinate arising from electric field induced symmetry breaking. After full structural optimization, the ground-state structure exhibits localized structural distortions, particularly in the region containing O and C impurity atoms. The calculated Berry-phase polarization for this structure is 5.75 μC/cm^2, which is consistent with the experimentally observed values. The structure clearly exhibits two stable polarized states separated by a non-polar centrosymmetric state, with a switching energy barrier of approximately 2.65 meV/f.u.

REFERENCES

(1) Giannozzi, P.; Baroni, S.; Bonini, N.; Calandra, M.; Car, R.; Cavazzoni, C.; Ceresoli, D.; Chiarotti, G. L.; Cococcioni, M.; Dabo, I.; Dal Corso, A.; De Gironcoli, S.; Fabris, S.; Fratesi, G.; Gebauer, R.; Gerstmann, U.; Gougoussis, C.; Kokalj, A.; Lazzeri, M.; Martin-Samos, L.; Marzari, N.; Mauri, F.; Mazzarello, R.; Paolini, S.; Pasquarello, A.; Paulatto, L.; Sbraccia, C.; Scandolo, S.; Sclauzero, G.; Seitsonen, A. P.; Smogunov, A.; Umari, P.; Wentzcovitch, R. M. QUANTUM ESPRESSO: A Modular and Open-Source Software Project for Quantum Simulations of Materials. *J. Phys.: Condens. Matter* **2009**, *21* (39), 395502. https://doi.org/10.1088/0953-8984/21/39/395502.

(2) Giannozzi, P.; Andreussi, O.; Brumme, T.; Bunau, O.; Buongiorno Nardelli, M.; Calandra, M.; Car, R.; Cavazzoni, C.; Ceresoli, D.; Cococcioni, M.; Colonna, N.; Carnimeo, I.; Dal Corso, A.; De Gironcoli, S.; Delugas, P.; DiStasio, R. A.; Ferretti, A.; Floris, A.; Fratesi, G.; Fugallo, G.; Gebauer, R.; Gerstmann, U.; Giustino, F.; Gorni, T.; Jia, J.; Kawamura, M.; Ko, H.-Y.; Kokalj, A.; Küçükbenli, E.; Lazzeri, M.; Marsili, M.; Marzari, N.; Mauri, F.; Nguyen, N. L.; Nguyen, H.-V.; Otero-de-la-Roza, A.; Paulatto, L.; Poncé, S.; Rocca, D.; Sabatini, R.; Santra, B.; Schlipf, M.; Seitsonen, A. P.; Smogunov, A.; Timrov, I.; Thonhauser, T.; Umari, P.; Vast, N.; Wu, X.; Baroni, S. Advanced Capabilities for Materials Modelling with Quantum ESPRESSO. *J. Phys.: Condens. Matter* **2017**, *29* (46), 465901. https://doi.org/10.1088/1361-648X/aa8f79.

(3) Giannozzi, P.; Baseggio, O.; Bonfà, P.; Brunato, D.; Car, R.; Carnimeo, I.; Cavazzoni, C.; De Gironcoli, S.; Delugas, P.; Ferrari Ruffino, F.; Ferretti, A.; Marzari, N.; Timrov, I.; Urru, A.; Baroni, S. Q UANTUM ESPRESSO toward the Exascale. *The Journal of Chemical Physics* **2020**, *152* (15), 154105. https://doi.org/10.1063/5.0005082.

(4) Resta, R. Electrical Polarization and Orbital Magnetization: The Modern Theories. *J. Phys.: Condens. Matter* **2010**, *22* (12), 123201. https://doi.org/10.1088/0953-8984/22/12/123201.

(5) Bhattarai, B.; Zhang, X.; Xu, W.; Gu, Y.; Meng, W. J.; Meng, A. C. Effect of Sc Spatial Distribution on the Electronic and Ferroelectric Properties of AlScN. *Mater. Horiz.* **2024**, *11* (21), 5402–5408. https://doi.org/10.1039/D4MH00551A.

(6) Perdew, J. P.; Burke, K.; Ernzerhof, M. Generalized Gradient Approximation Made Simple. *Phys. Rev. Lett.* **1996**, *77* (18), 3865–3868. https://doi.org/10.1103/PhysRevLett.77.3865.
